# Gender Representation in Academic Publications of Tourette Syndrome Research: An Analysis of Authorship Trends

**DOI:** 10.7759/cureus.51520

**Published:** 2024-01-02

**Authors:** Akanksha Mahajan, Vaishnavi K., Neha Dikshit, Jasreen Kaur Sandhu, Lalitha Lalithya Pallempati, Laura Olivieri

**Affiliations:** 1 Medical School, Government Medical College, Amritsar, Amritsar, IND; 2 Medical School, Sapthagiri Institute of Medical Science and Research Centre, Bengaluru, IND; 3 Pediatrics, Government Medical College, Miraj, Miraj, IND; 4 Nephrology, Government Medical College, Amritsar, Amritsar, IND; 5 Psychiatry, Kamineni Academy of Medical Sciences and Research Centre, Hyderabad, IND; 6 Internal Medicine, University of New England College of Osteopathic Medicine, Biddeford, USA

**Keywords:** gender trends, first author, publication equality, gender gap, tourette syndrome

## Abstract

Tourette syndrome, characterized by phonic and motor tics, is a neurodevelopmental disorder that significantly affects the quality of life of people with the condition. With over 1500 articles published in the last decade alone, this study aims to analyze the gender trends in publications related to Tourette syndrome from 2013 to 2022, examining the number of female authors as first authors and predicting future changes in female participation. The aim of this study is to analyze the gender trends of the first author in publications related to “Tourette Syndrome” from PubMed-indexed publications from January 2013 to December 2022. A bibliometric analysis was conducted by the search engine PubMed for articles pertaining to “Tourette Syndrome”. NamSor app V.2 (Namsor™ Applied Onomastics, NamSor SAS, Versailles, France), an application programming interface (API), was used to identify the gender of the first publishing author. Statistical analysis and graphic models were produced using R software (R Foundation for Statistical Computing, Vienna, Austria), ARIMA (Autoregressive Integrated Moving Average) model, and DataWrapper (Datawrapper GmbH, Berlin Prenzlauer Berg, Germany). Based on the analysis of 1052 publications on Tourette Syndrome, it was found that 54.8% (n=576) of the first authors were females. A significant association was observed between gender ratios and country of publication. Statistical forecasting models suggest that female participation in Tourette research is expected to rise to approximately 60 by the year 2027. Therefore, the study highlights the progress made in achieving gender parity in Tourette syndrome research, with female-led publications being on par with male-led publications. However, there is still a need to address the global gender gap and geographical disparities in research to work towards a more inclusive and diverse academic environment.

## Introduction and background

Tourette syndrome, characterized by phonic and motor tics, is a neurodevelopmental disorder that significantly affects the quality of life of people with the condition [1,2,3]. Although common, Tourette syndrome is a challenging disease to treat as its underlying pathophysiology is not thoroughly understood [4]. As of today, a vast array of treatment modalities is available to control the multitude of symptoms and associated behavioral comorbidities like attention deficit hyperactive disorder (ADHD) and obsessive-compulsive disorder (OCD), ranging from simple drug therapy with tetrabenazine to more targeted treatment with deep brain stimulation [2,3].

Academic publications play a crucial role in career advancement and provide quality information for future trainees and medical professionals [5]. Gender trends in academics, particularly in the health sector, have been under the radar in recent times, focusing on opportunities and major changes aiding women authorships. Although there is a rising trend in female authorship worldwide, men continue to dominate the field [5]. This is due to reasons such as inequalities in grants and scholarships for conducting research, the establishment of labs, publication support and funding, and placement and job availability [6,7]. Another important contributing factor is the time constraint as women are unable to give as much time as their male counterparts to longer cohort studies that take years for analysis [6,7].

The aim of this study was to determine the number of female authors contributing as first authors in publications related to Tourette syndrome from January 2013 to December 2022 and to compare it against the total number of first authors in the same period, by using bibliometric analysis. An attempt has been made to analyze whether there was a rise, fall, or steady state in female contribution as first authors and to predict the change in the coming decade with regard to the overall participation of women as first authors.

## Review

This is a bibliometric analysis conducted on May 9th, 2023. The analysis was carried out using the search engine PubMed. Articles pertaining to “Tourette Syndrome” were analyzed, to ascertain the gender of the first author, to find out gender trends, and to predict the upcoming trends. PubMed was searched for the term “Tourette Syndrome.” The last 10 years of articles were analysed from 1st January 2013 to 31st December 2022. Publications from the year 2023 were excluded since only a few publications of 4 months were available. However, articles accepted in the year 2022 and published and appearing on PubMed in 2023 were included in the study.

All the relevant articles were downloaded as a Microsoft Excel file (Microsoft Corporation, Redmond, USA). There were a total of 1054 entries which were equally divided among the six authors of this study. Each author scrutinized the article for its relevance to the aim of our study. All the data was entered into a Google spreadsheet (Google Inc., Mountain View, USA). Each entry was completed to include PubMed identifier (PMID), title, citation, journal, year of publication, and direct object identification (DOI). Then subsequently the full name (first and last name), country, and university/institute of the first author were completed via PubMed. Only binary genders (Male and Female) were taken for the purpose of this study. The full name and country (where publication was done) of the first author were used to find out their gender. Gender analysis was limited to first authors only because they contribute more to the paper and are generally the ones to come up with the idea.

NamSor app V.2 (Namsor™ Applied Onomastics, NamSor SAS, Versailles, France), an application programming interface (API), was used for this study [8]. NamSor was selected for gender identification because of its large dataset (7.5 billion names); also, it uses both name and country of origin for predicting gender. NamSor has a proven accuracy of 97%, is partially free to use, and is easy to learn for beginners [9]. Quality inferential is also provided by NamSor, which was not used for this study. To settle any discrepancies a simple internet search was carried out to confirm the gender.

Statistical analysis was done using R software version 4.3.2 (R Foundation for Statistical Computing, Vienna, Austria), and the ARIMA (Autoregressive Integrated Moving Average) model and graphs were prepared using DataWrapper (Datawrapper GmbH, Berlin Prenzlauer Berg, Germany).

Result

**Figure 1:**
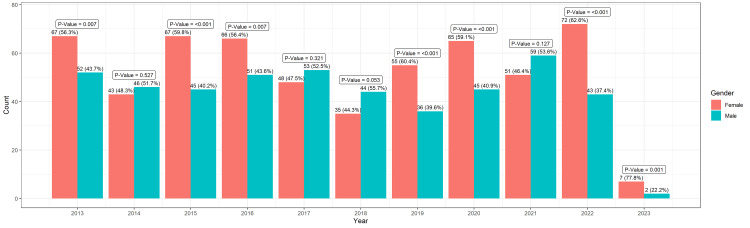
The number of male and female first authors each year. Binomial Test is used. p <0.05 is significant.

A total of 1054 studies were taken into consideration, and out of those, two were excluded as the full name of the first author could not be found. Out of the remaining 1052 studies, 576 had females as first authors, i.e., 54.8%, and 476 had males as first authors, i.e., 45.2%. Figure 1 shows the number of male and female authors each year. The maximum number of female first-author publications on “Tourette Syndrome” was in the year 2022. A statistically significant difference (p <0.05), with more number of females as first authors compared to male first authors was observed in the years 2013, 2015, 2019, 2020, and 2022.

**Figure 2:**
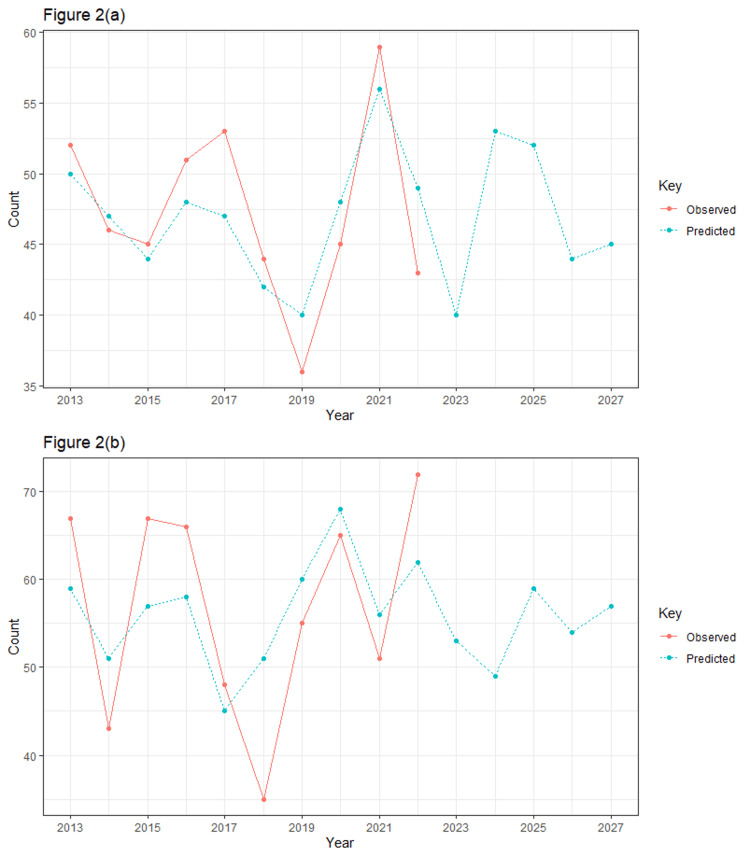
(a) Prediction of publication trends for male first authors; (b) prediction of publication trends for female first authors for the next 5 years/10 years.

Figure 2a shows the prediction of publication trends for male first authors and Figure 2b shows the prediction of publication trends for female first authors for the next 5 years/10 years. It is expected that in the year 2027, publications with males as first authors would be around 45 and those with female first authors would be close to 60. The statistical model used is the ARIMA model. The modelling data is taken from 2013 to 2022. The year 2023 is not used to model, because full data was not available for it. The forecasting is done from 2013 to 2032.

**Figure 3:**
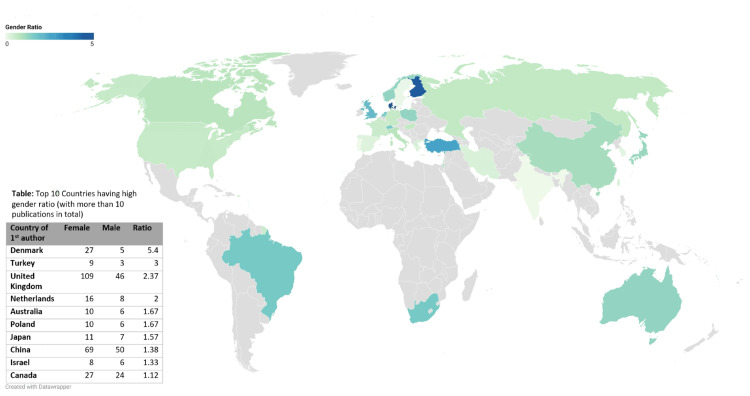
Gender trends in publications based on the country from 2013-2023. Denmark was found to have the maximum publications with females as first authors, and a gender ratio of 5.4. The countries with more than 10 publications in total were considered.

Figure 3 shows gender trends in publications based on the country from 2013-2023. Denmark was found to have the maximum publications with females as first authors and a gender ratio (female:male) of 5.4. The countries with more than 10 publications in total were considered. Fisher's Exact Test was performed for the gender and country variables. We got a p-value <0.001. This signifies that there is a significant association present between gender and country.

Table 1 shows the top 10 journals having a favorable gender ratio (with more than 10 publications in total).

**Table 1:** Top 10 journals having a favourable gender ratio (with more than 10 publications in total). F1000Res: F1000Research; Neurosci Biobehav Rev: Neuroscience & Biobehavioral Reviews; Biol Psychiatry: Biological Psychiatry; Front Neurol: Frontiers in Neurology; Tremor Other Hyperkinet Mov (N Y): Tremor and Other Hyperkinetic Movements; Brain Sci: Brain Sciences; J Psychiatr Res: Journal of Psychiatric Research; Front Psychiatry: Frontiers in Psychiatry; Behav Neurol: Behavioural Neurology; Psychiatry Res: Psychiatry Research; Front Neurosci: Frontiers in Neuroscience; J Child Neurol: Journal of Child Neurology; Sci Rep: Scientific Reports; J Neuropsychol: Journal of Neuropsychology

Journal/Book	Female	Male	Ratio
F1000Res	6	7	0.86
Brain	7	8	0.88
Neurosci Biobehav Rev	13	12	1.08
Biol Psychiatry	8	7	1.14
PLoS One	7	6	1.17
Front Neurol	13	11	1.18
Tremor Other Hyperkinet Mov (N Y)	6	5	1.2
Brain Sci	9	7	1.29
Cortex	12	8	1.5
J Psychiatr Res	8	5	1.6

Table 2 shows the Top 10 journals having a high gender ratio (with more than 10 publications in total).

**Table 2:** The Top 10 journals having a high gender ratio (with more than 10 publications in total.) F1000Res: F1000Research; Neurosci Biobehav Rev: Neuroscience & Biobehavioral Reviews; Biol Psychiatry: Biological Psychiatry; Front Neurol: Frontiers in Neurology; Tremor Other Hyperkinet Mov (N Y): Tremor and Other Hyperkinetic Movements; Brain Sci: Brain Sciences; J Psychiatr Res: Journal of Psychiatric Research; Front Psychiatry: Frontiers in Psychiatry; Behav Neurol: Behavioural Neurology; Psychiatry Res: Psychiatry Research; Front Neurosci: Frontiers in Neuroscience; J Child Neurol: Journal of Child Neurology; Sci Rep: Scientific Reports; J Neuropsychol: Journal of Neuropsychology

JournalBook	Female	Male	Ratio
Front Psychiatry	23	7	3.29
Behav Neurol	13	5	2.6
Psychiatry Res	19	8	2.38
Front Neurosci	11	5	2.2
J Child Neurol	14	7	2
Sci Rep	8	4	2
J Neuropsychol	7	4	1.75
J Psychiatr Res	8	5	1.6
Cortex	12	8	1.5
Brain Sci	9	7	1.29

Discussion

The present study was conducted by taking into consideration 1052 studies about Tourette syndrome in the PubMed index portal. The gender associated with the first author and the affiliation with the university’s country were taken into consideration. The search for the gender trends in the publication of Tourette syndrome indicated that out of these 1052 studies, 576 had females as first authors, i.e., 54.8%, and 476 had males as first authors, i.e., 45.2%.

Past studies on gender trends in publication in different journals have suggested that the percentage of female authors in *The Lancet Global Health* and other journals is low compared to male authors [10,11]. Due to the influence of unfair systems that continue to disfavour women, this is apparent. Women find it hard to receive grants as compared to men causing a greater gap in publications [12,13]. Influenced by historical and systemic biases, research institutes and organisations add to limit their growth in the progression of academia. There is evidence that factors like patriarchal, racialised, and colonial systems also play a very significant role in who publishes articles [14,15].

The current analysis focuses on a gender study of Tourette syndrome, which outperforms the general gender publishing trends on a few important metrics. This study strengthens the case for the need for demands for inclusive career structures that are fair and equitable, with research and educational organizations dispersed equitably over the globe. The data in our study indicates that Denmark, Turkey, and the United Kingdom are home to most of the first authors. When compared with general medicine publications, specialized journals for global health were shown to have a greater publishing gender ratio. These findings may be of the different article types produced by the different journals but indicative of the gender publication trends on a deeper level. It is also revealed that location, publication characteristics, and impact journals played a very important role in the gender ratio.

Promoting gender equality in Tourette syndrome research involves raising awareness and offering education, supporting female researchers through career development and funding, fostering networking and mentorship, implementing policies for equal opportunities, collecting and disclosing gender-related data, and addressing geographic imbalances through international cooperation and equitable funding distribution.

This study was subjected to several limitations. Firstly, the country mentioned in affiliation was taken into consideration in its determination of gender with Namsor. There is some level of measurement error with respect to the gender-neutral identifying authors. Secondly, in analysing the author's gender, the author's affiliation was considered with the university’s country, which might lead to an overestimation of female authors. Thirdly, this study just considered the first author of each paper, which may lead to a significant gap in the gender study. And lastly, only PubMed was used; other search engines can also be included.

## Conclusions

In conclusion, female author-led publications are on par with male author-led publications vis-a-vis Tourette syndrome. In a global setting, the European countries have shown significantly higher female first authors. It is imperative to bridge this gap worldwide pertaining to every field of medicine. It is suggested that further analysis is to find a detailed account taking into consideration the structural biases, geographical locations, grant access, and all the contributing authors. 
